# Management of thoracoabdominal gunshot injuries by using minimally invasive surgery at role 2 deployed field hospitals in Ukraine

**DOI:** 10.1186/s12893-024-02475-3

**Published:** 2024-06-14

**Authors:** Igor Lurin, Oleh Vorovskiy, Vitalii Makarov, Eduard Khoroshun, Volodymyr Nehoduiko, Andrii Ryzhenko, Stepan Chobey, Maksym Gorobeiko, Andrii Dinets

**Affiliations:** 1 National Academy of Medical Sciences of Ukraine, Kyiv, Ukraine; 2 State Institution of Science “Research and Practical Center of Preventive and Clinical Medicine”, State Administrative Department, Kyiv, Ukraine; 3 National Pirogov Memorial Medical University, Vinnytsya, Ukraine; 4 Department of Thoraco-Abdominal Surgery, Military Medical Teaching Center of the Northern Region of Ministry of Defense of Ukraine, Kharkiv, Ukraine; 5 Department of Surgery #4, Kharkiv National Medical University, Kharkiv, Ukraine; 6 Military medical clinical center of the central region, Vinnytsya, Ukraine; 7 Department of Surgery, Uzhgorod National University, Uzhgorod, Ukraine; 8 Department of Healthcare, Faculty of Postgraduate Education, Kyiv Agrarian University, Kyiv, Ukraine; 9 Department of Surgery, Lancet Clinic and Lab, Kyiv, Ukraine; 10 Department of Surgery, Verum Expert Clinic, vul. Demiїvska 13, Kyiv, 03039 Ukraine

**Keywords:** Thoracoabdominal injury, Chest war injury, Abdomen war injury, Video-assisted thoracoscopy, Minimally invasive surgery, Russia-Ukraine war, Russo-ukrainian war

## Abstract

The Russia-Ukraine war is associated with critical and severe thoracoabdominal injuries. A more specific approach to treating patients with thoracoabdominal injury should also include minimally invasive technologies. It remains unclear about the utility of using video-assisted thoracoscopic surgery (VATS) and laparoscopy in patients with thoracoabdominal injury. The aim of this study was to investigate and evaluate the utility of video-assisted thoracoscopic surgery, laparoscopy as well as magnetic tool applications for the management of severe thoracoabdominal injury in combat patients injured in the ongoing war in Ukraine and treated in the Role 2 deployed hospital. **Patients and methods** 36 male combat patients thoracoabdominal injury were identified for the study during the first 100 days from February, 24 2022. These individuals were diagnosed with thoracoabdominal GSW in the Role 2 hospital (i.e. deployed military hospital) of the Armed Forces of Ukraine. Video-assisted thoracoscopy surgery (VATS) and laparoscopy with application of surgical magnetic tools were applied with regards to the damage control resuscitation and damage control surgery. **Results** In 10 (28%) patients, VATS was applied to remove the metal foreign body fragments. Both thoracotomy and laparotomy were performed in 20 (56%) hemodynamically unstable patients. Of these 20 patients, the suturing of the liver was performed in 8 (22%) patients, whereas peri-hepatic gauze packing in 12 (33%) patients. Massive injury to the liver and PI 2.0–3.0 were diagnosed in 2 (6%) patients. Lethal outcome was in 1 (2.8%) patient. **Conclusions** Thoracoabdominal gunshot injuries might be managed at Role 2 hospitals by using video-assisted thoracoscopy (VATS) and laparoscopy accompanied by surgical magnetic tools. Damage control surgery and damage control resuscitation must be applied for patients in critical and severe conditions.

## Introduction

The ongoing Russia-Ukraine war is associated with critical and severe injuries of military personnel and civilian population due to the frequent application of all possible kinds of high-energy weapons, resulting in severe injuries to military personnel and civilians [1–8]. Injuries to the thorax and abdomen are on the top among other sites of gunshot traumas in warfare and armed conflicts and are frequently presented as combined thoracoabdominal injuries. The latter is associated with a high rate of lethal outcomes and longer rehabilitation in those, who survived due to injuries of multiple organs and major vessels of the chest and abdomen, bone fractures as well as severe maxillofacial trauma [9–12]. The modern weapon is designed to cause critical and severe damage in multiple organs which is presented by separate injuries to the chest or abdomen, or combined trauma, including abdominal and thoracoabdominal cases [1, 5, 6, 13, 14].

Thoracoabdominal injury is considered a combination of abdominal injury with chest and diaphragm damage [2]. Thoracoabdominal injury due to high-energy weapon application is always associated with lung failure, hemo- and pneumothorax, hemorrhagic shock, cardiac tamponade, and upper airway obstruction with blood and mucus due to post-traumatic hypersecretion of mucous membranes. On the other hand, the abdominal trauma is also severe, presenting with injury to the liver, intestine, or mesenteric and other major vessels, resulting in massive hemorrhage and peritonitis. Thoracoabdominal injury is also frequently associated with diaphragm damage, which might be hidden. The so-called “hidden” damage to the diaphragm is especially difficult to establish, however, it is usually suspected at the preoperative stage as well as intraoperatively. The most common clinical signs of a rupture of the diaphragm are considered to be the presence of a gunshot wound entrance below the V rib, bruises and fractures of the ribs in the same location, decreased breath sounds, sounds of intestinal peristalsis during lung auscultation, or signs of intestinal obstruction. However, in combat patients, the classical signs of damage to the diaphragm are not always detected or are hidden by the clinic of injuries to other organs. The management of patients with thoracoabdominal injury should include the application of Advance Trauma Life Support (ATLS) protocol, focused assessment with sonography in trauma (FAST) protocol as well as computed tomography of chest and abdomen. Surgical management is envisaged application of damage control surgery, whereas recent studies also suggested damage control resuscitation for severe combat trauma [11, 15, 16]. A more specific approach to treating patients with thoracoabdominal injury should also include minimally invasive technologies to reduce surgical trauma for patients in critical and severe conditions, which is frequent in war settings. In the ongoing war in Ukraine, thoracoabdominal injuries constituted 0.5% out of all cases. The possibility of application of minimally invasive technologies (i.e. laparoscopy and thoracoscopy) in the Role 2 hospitals (i.e. deployed military hospital) in the ongoing Russo-Ukrainian war is associated with multiple technical and safety issues, due to high risk of blackouts, problems with supplies for the generators as well as high risk of cruise-missiles or multiple rocket launch rockets attacks on the civil or military medical facilities by russia army [1, 2, 17, 18]. The little is known about the application of laparoscopy and thoracoscopy in the Russo-Ukrainian war, indicating need for further evaluation of video-assisted thoracoscopic surgery (VATS) and laparoscopy over the open surgical techniques in patients with severe trauma at Role 2 hospitals.

The aim of this study was to investigate and evaluate the utility of laparoscopy, video-assisted thoracoscopic surgery and magnetic tool applications, as well as to demonstrate the possibility of using minimally invasive approach (laparoscopy and thoracoscopy) for the management of severe abdominal and thoracoabdominal injuries in combat patients injured in the ongoing war in Ukraine and treated in the Role 2 hospital.

## Patients and methods

A 36 male combat patients were identified for the study during the first 100 days from February, 24 2022. These individuals were diagnosed with thoracoabdominal GSW at the Role 2 hospital of the Armed Forces of Ukraine in Donetsk oblast. The exact number of all injured individuals for the mentioned period can not be shown at the time of manuscript submission because these data are classified. The medical roles system of military care in Ukraine was presented in our previous publications [2, 4, 5, 19, 20].

The thoracoabdominal injury was considered severe for cases with an Injury Severity Score (ISS) ≥ 16 or for cases presenting with two or more injured body areas, which corresponded to the score ≥ 3 according to the Abbreviated Injury Scale (AIS) [21]. Upon admission, all injured were evaluated by clinical chemistry blood tests, and ECG. An ultrasound examination was performed according to the extended Focused Assessment with Sonography for Trauma (e-FAST) protocol using a portable ultrasound machine “Sonosite Micromaxx, 2017”, equipped with probes having the Doppler and M-mode for the 2D visualizations. Software SonoCalc was used for the ultrasound measurements of the trauma extent. X-ray examination was performed by using the machine “Opera RT20, 2018”, which is equipped with modules to have contact with a good focus on the patients in a distance of 150–180 cm, which is specifically important for patients with severe gunshot injury. Digital X-ray surgical device type C-arc was applied as well (“Siemens Siremobil Compact, 2017”). A computed tomography (CT) scan was performed by using the “Planmed Verity, 2019” machine equipped with modules for 3D visualization and patient positioning. All patients underwent catheterizations of the subclavian vein, bladder, and nasogastric intubation. Video-assisted thoracoscopy surgery (VATS) was performed by using the system “Richard Wolf, 2011”. Two minimally invasive towers for laparoscopy and VATS were available: one from the civil hospital to be turned into Role 2 hospital and other one from the military medical team, indicating good cooperation approach between civil and military medicine. The indications for the surgery were positive peritoneal signs, free fluid and air in the abdominal cavity, presence of the foreign bodies with a greatest diameter > 1 cm. Thoracotomy was performed in case of continuing bleeding, open hemothorax, clotted hemothorax, air leak. The surgery team were two surgeons, surgery nurse, anesthesiologist and anesthesiology nurse. All patients underwent thoracocentesis with subsequent application of surgical suction drainage to the pleural cavity. Thoracotomy was performed for patients with bleeding over 250 ml/hour or immediate receiving of > 1,200 ml of blood by surgical suction drainage. All patients who did not met above-mentioned criteria were operated by VATS and laparoscopy. The inclusion criterion was performing VATS and laparoscopy, all other patients were excluded from this study. To preserve CO_2_ in the unsealed abdominal cavity in the conditions of penetrated gunshot injury, we temporarily sutured inlet/outlet projectiles holes, or use it as a port for the instruments. The gas pressure was considered as sufficient in the abdominal cavity at the level of 12–14 mm of Hg. Gas insufflation is carried out into the abdominal cavity. In case of minimally invasive approach to pleural cavity the gas insufflation was not performed, because of the separate intubation of the lungs. The surgical magnetic tools were designed and manufactured in Ukraine [22].

The routine protocol for surgical removal of ferromagnetic fragments using VATS and magnetic tools was shown previously [2, 22]. In brief, the foreign body (metal fragment) was fixed by a surgical magnetic tool in a lateral position. To reduce the risk of secondary injury to the adjacent fragment tissues while removing a fixed foreign body, the foreign body needed to be located on the same axis as the magnetic tool. The rotation and fixation of the metal foreign body (bullet or shell etc.) was performed with the assistance of the magnetic multifunctional tool for the diagnosis and removal of metal ferromagnetic foreign bodies [2, 22].

A perfusion index (PI) was calculated for all patients and PI values were considered in surgical planning. Damage control resuscitation as a part of damage control surgery (DCS) was applied for patients in the case of PI within the range of 2–3%. Management of the shock was performed until the stable hemodynamics. Patients underwent completed surgical interventions in case of achieving normal PI > 4%, which was considered as sufficient result after the DCR. The above-mentioned tactics of DCS with damage control resuscitation were quickly adopted in our routine clinical war surgery settings with the general title of “Damage control reanimation” (DCR). DCR is an emergency method, applied for patients with severe post-traumatic hemorrhagic shock. The patients with such injuries are managed by the simultaneous application of reanimation and surgical operation upon admission to the Role 2 hospital, which resulted in the adoption of the integral conception of DCR/DCS. The concept of DCR/DCS was adopted by Ukrainian military surgeons only for patients in critical conditions in order to do the reanimation measures upon the admission of the patients without delaying the surgical interventions, which resulted in better survival of the patients [20, 23].

## Results

All patients were male with a mean age of 32 years (range 19–54 years). In 32 (89%) of the patients were diagnosed with right-side injuries to the chest and the lift-side in 24 (67%) patients. Analyses of the cohort showed the following injuries in addition to the thoracoabdominal GSW: fractures of ribs in 21 (58%), scapula in 1 (3%) pericardium wounds in 1 (3%) patient. Severity injury of the patients was moderate in 14 (39%) patients (ISS < 16), severe in 33 (92%) patients (ISS 16–25), critical in 9 (25%) patients (ISS > 25), and lethal outcome was in 1 (3%) patient due to.

The mean time of surgical operation was 50 ± 5.2 minutes (the time from the incision to the final stich”, excluding intubation and extubating time). The application of endoscopic methods did not increase the duration of the surgery. The “conversion by demand” was performed immediately upon the evaluation of the injury severity and the patient’s condition.

Surgical suction drainage (i.e. Bülau drain) was applied for all 36 (100%) patients within 10 ± 0.5 min upon admission, followed by transporting the patient to the operation room for DCR and surgical management. The FAST protocol was applied for all patients to check for possible hemoperitoneum and hemopericardium, showing effectiveness in 30 (83%) patients, whereas false-positive detection of the fluid was detected in 6 (17%) patients. Out of these 6 patients, the misdiagnosis was detected in 3 (8.3%) patients, presenting with clotted pneumothorax in 2 (6%) cases, and non-diagnosed hemoperitoneum in the lower pelvis in 1 (3%) case. A CT scan was applied in 18 (50%) patients and an X-ray in all 36 (100%).

Hemopneumothorax was diagnosed in 36 (100%) patients. These patients were admitted with previously placed surgical suction drainage, however in 18 (50%) patients we had to replace it. The derange replacement was performed due to unreliable fixation, the insufficient diameter of the tube, and the placement of drainage out of the pleural cavity. The hole created for the thoracentesis was used as a port for thoracoscopy in 10 (28%) patients.

Further data analyses showed that 2 (6%) patients had a massive injury to the liver along with the PI 2–3%, resulting in a two-step approach of simultaneous surgical operation (with limited surgical extent) and application of DCR. The completed surgical operations were performed for these 2 patients after the stabilization of their hemodynamics as well as PI > 4%. Other 34 (96%) patients were presented with PI > 4%, indicating good hemodynamics and possibilities to apply VATS and laparoscopy. Autologous blood transfusion was performed in 17 (47%) patients, other patients were resuscitated using crystalloid fluids infusions.

VATS was performed in all 36 patients. Of these 36 patients, in 10 (28%) patients, VATS was applied to remove the metal foreign body fragments, remove coagulated hemothorax, suturing of the lung wounds as well and apply bipolar electrocautery to the liver wounds using GSW defect in the diaphragm as a port to the abdominal cavity. The diaphragmatic wounds were also sutured under the conditions of VATS.

**Fig. 1:**
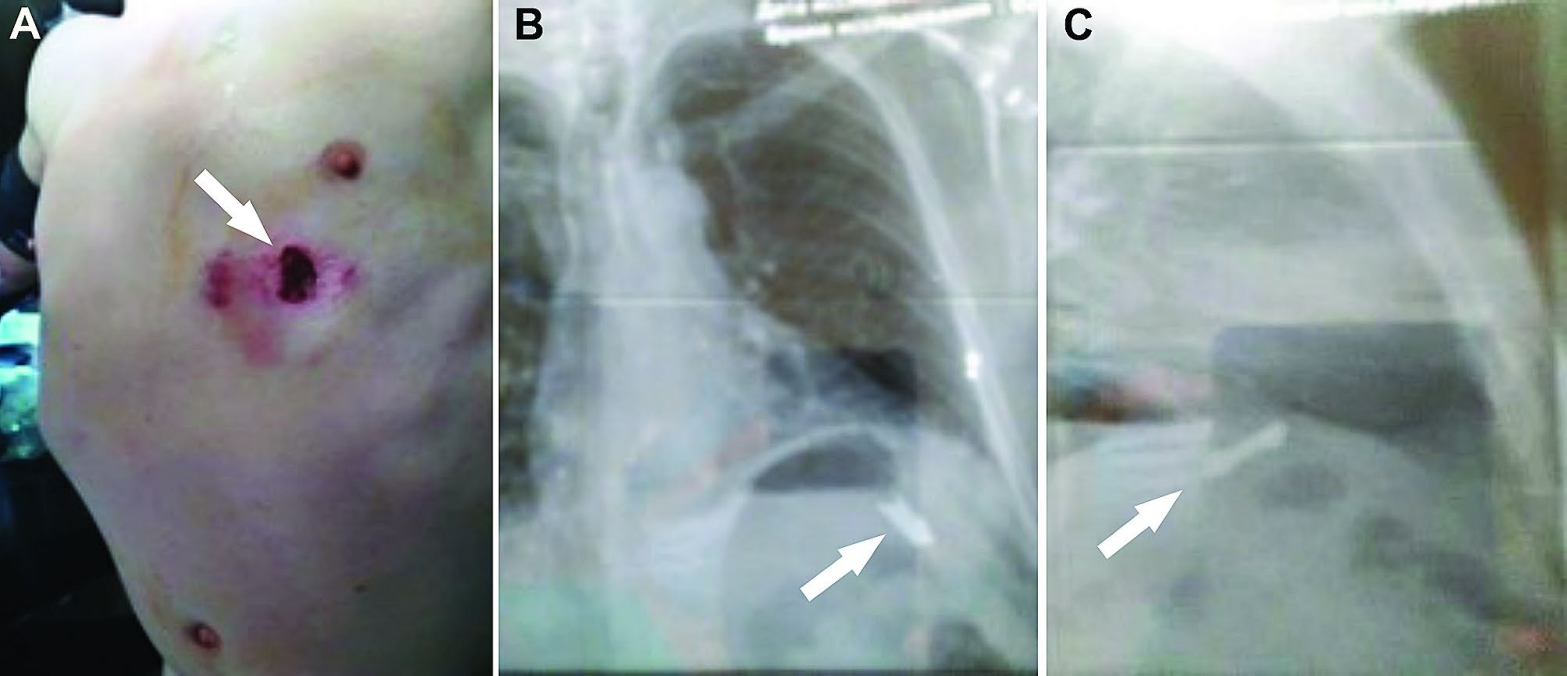
Illustration of the patient with thoracoabdominal gunshot injury. (**A**) Intraoperative photograph showing inlet projectile hole 3,0 × 2.5 см in the 5 left intercostal area on midclavicular line. Anterior (**B**) and lateral (**C**) view of the chest X-ray film with signs of metal foreign body in the left subdiaphragmatic area (marked with arrow)

**Fig. 2:**
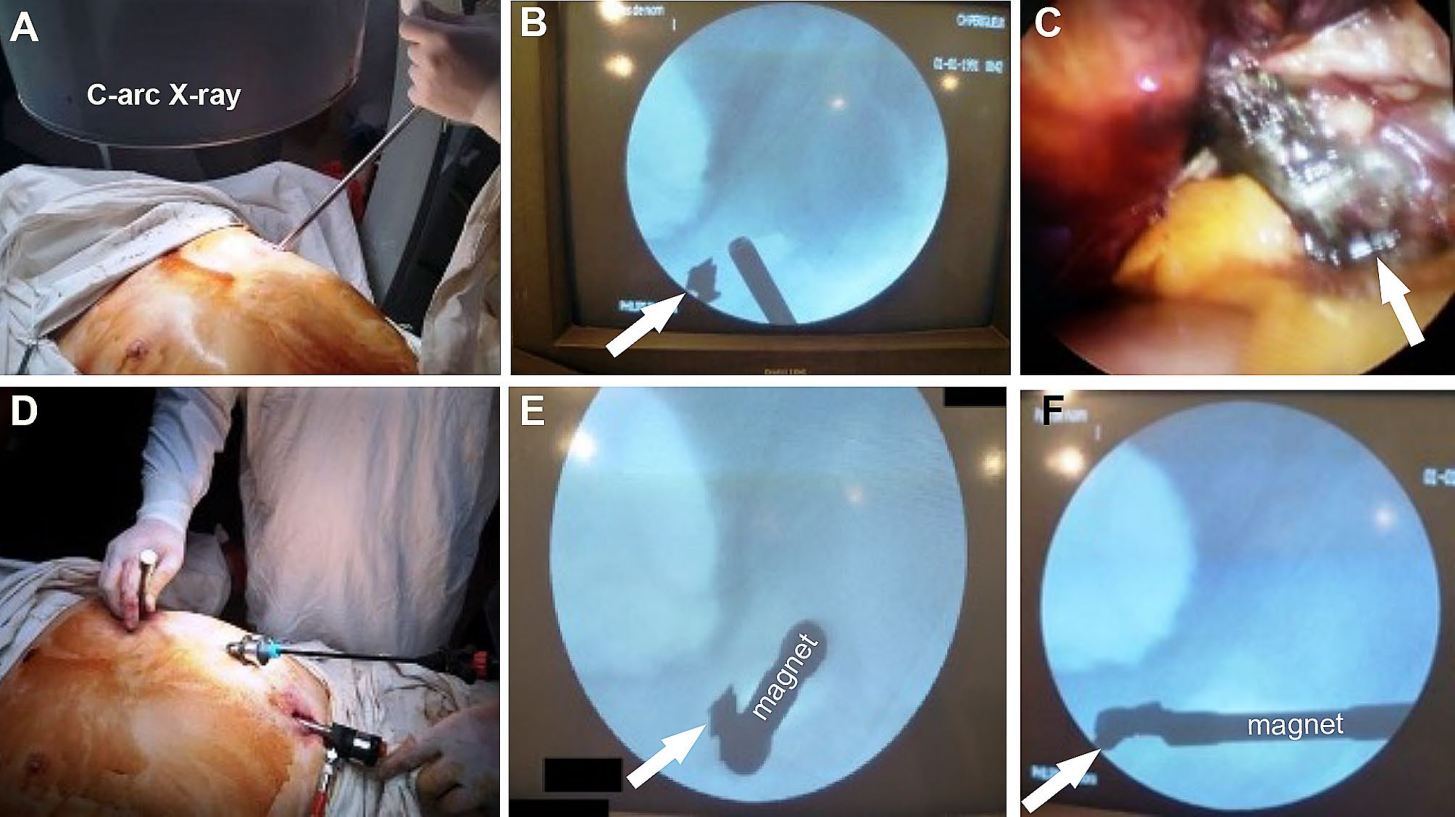
Intraoperative photograph of the video-assisted thoracoscopy (VATS) and laparoscopy procedures. (**A)** A thoracoscope was placed into the thoracic cavity using an inlet hole as a port to perform the revision and revealed no evidence of the metal fragment in the chest. (**B)** Intraoperative X-ray view by C-arc showing the presence of the metal fragment (marked with arrow) in the abdomen. (**C)** Intraoperative laparoscopy image showing metal fragment (marked with an arrow) within the S2 segment of the liver. (**D)** Illustration of the magnetic tool to be inserted into the abdomen (held by the right hand of the surgeon) through the inlet hole in the chest under the laparoscopy guidance as preparation steps to remove the metal fragment from the S2 segment of the liver. (**E)** X-ray view of the fixed metal fragment (marked with an arrow) by the magnetic tool to further perform its rotation. (**F)** X-ray view of the rotated metal fragment (marked with an arrow) to the lateral position by the magnetic tool

**Fig. 3:**
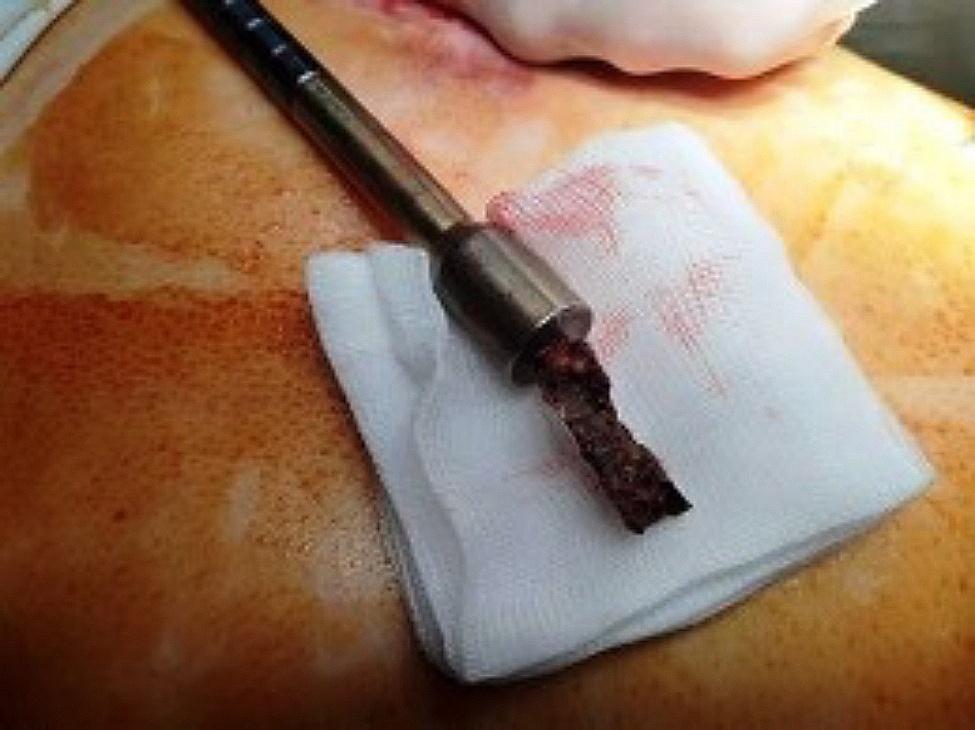
Photograph of metal fragment 3.5 × 2.3 cm fixed by the magnetic tool after the removal from the abdomen

A typical case of the patient with thoracoabdominal injury is illustrated in Fig. 1. Simultaneously with VATS, the laparoscopy was applied for 32 (89%) patients, presenting with severe liver bleeding, whereas in 4 (11%) patients, the conversion to the laparotomy was performed due to severe continuing bleeding that cannot be stopped by minimally invasive surgery. Of these 32 patients with simultaneous VATS and laparoscopy, in 28 (78%) patients, the bleeding from the liver was stopped by applying bipolar electrocautery, whereas in 4 (11%) patients the wounds were sutured because of the wound defect length of 3 to 5 cm was associated with continuing bleeding. Laparoscopy for right side injuries was performed for 30 (83%) patients as well as for left side injuries in 6 (17%) patients, indicating the right side to be more frequent. The metal fragments from the gunshot projectiles were found in the diaphragm and associated with superficial injury to the liver in 4 (11%) patients and 32 (89%) patients we identified more severe wounds to the liver by metal fragments. The analyses of these 32 patients showed one metal fragment in 28 (78%) patients, two in 3 (8.3%) patients, three in 2 (6%) patients, four in 1 (3%) patient, five in 1 (3%) patient, six in 1 (3%) patient. These metal fragments from the shelling were removed laparoscopically in 13 (36%) patients. The multifunctional magnetic tool was applied to remove foreign bodies from the pleural or abdominal cavity was used in 10 (17.8%) patients, whereas another magnetic tool designed for the video-endoscopic approach was applied in 13 (23.2%) patients. The application of a magnetic tool for the removal of metal fragments was performed in 20 (35.7%) patients under X-ray guidance by using a C-arc device (Fig. 2). The rotation and fixation of the metal fragments were performed laparoscopically in 5 (13.9%) patients. The size of the removed metal fragments varied from 2 × 1.2 cm to 4.3 × 2.8 cm (Fig. 3). The specific feature of the abovementioned operations using advanced surgical features was their application in conditions of the massive admission of injured patients to Role 2 hospitals. The rubber tubes were used to drain chest, left infradiaphragmatic space, abdominal cavity, subheptic space, pelvic space. We usually placed 1 drain to chest, at least 2 drains to left part of abdomen and at least 3 drain tubes to the left part. The exact evaluation of draining approach was not performed for this study. All patients demonstrated both stabile hemodynamics and respiration (usually 2–3 days postoperatively) were transported to higher echelon of medical care to continue treatment, and the follow up data is not available. The time of transportation to the hospital of higher Role depended on the combat situation with a specific consideration of drones attacks, ballistic and artillery strikes by the russian army to the sanitary transport, which is frequent.

The microbial contamination was considered, thus all patients received antimicrobial therapy by broad-spectrum antibiotics at Role 2 hospital.

## Discussion

In this study, we reported our clinical experience in the management of abdominal and thoracoabdominal GSW with the application of such minimally invasive technologies as VATS and laparoscopy at Role 2 hospitals in the ongoing Russo-Ukrainian war. We would like to stress the fact, that such approach is in contrast to the current NATO guidelines for the Role 2 medical aid, suggesting evacuation of the severe patients to the hospital of higher Role by helicopters. However, reality of Russo-Ukrainian war to suggest avoid any aircraft using for the medical purposes considering extremely high chances of the shutting down such vehicles by the enemy’s stationary missiles. Considering this experience, all patients are transported by the ground and in case the high risk for ground transportation, we have to change the approach for the medical aid, resulting in the decision to bring minimally invasive surgical equipment to the Role 2 hospitals (20–40 km from frontline), despite the risk for the surgical team and this equipment being destroyed by Russian attack, which is frequent to the medical facilities. To our best knowledge, this is the first study reporting a relatively large cohort of combat patients who underwent management of abdominal and thoracoabdominal gunshot wounds by using laparoscopy and VATS with surgical magnetic tools application. Previously, the minimally invasive technologies were presented only as case reports or during the hybrid period of russian aggression [2, 24, 25]. Another important message from this study is to show the possibility of routine application of the above-mentioned minimally invasive tools under the conditions of the attacks of high-energy weapons like MLRS, drones, and cruise missiles by the russian army. We also consider this study as evidence of the possibility of applying minimally invasive surgery and the surgical magnetic tools at the low echelons of medical care (Role 2 hospital is usually deployed 20–40 km from the battlefield line), which is important, considering the frequent violation of humanitarian law by the russian army as well as under the conditions of limited medical resources in Ukraine [1, 17, 26, 27]. Limited medical resources were and remain a common problem for healthcare in Ukraine due to various causes, including bad planning [12, 18, 28]. However, military surgeons demonstrated a high ability to make appropriate clinical decisions for patients with a severe thoracoabdominal injury even in unstable combat conditions and available resources at various echelons of medical care, including Role 2 hospitals.

Findings from this study support our hypothesis that VATS and laparoscopy are useful minimally invasive methods for the management of severe thoracoabdominal gunshot injury in war settings [2, 24]. Our results also suggest laparoscopy as an important part of the diagnosis and treatment of severe thoracoabdominal gunshot injury, which is in line with other studies of combat injury [29, 30]. It is well known that laparoscopy is associated with lower surgical trauma as compared to open surgery, which is important for patients already having severe injuries. On the other hand, laparoscopy might be considered a routine procedure for combat patients in case of absence of massive admission of the patients to Role 2 hospitals. Other limitations of the laparoscopic interventions are obvious highly traumatic lesions to the abdomen, eversions, and hemodynamically unstable patients. For these patients, we immediately perform laparotomy to stop bleeding, excise necrotic tissues, as well as clean the abdomen from dirt and foreign bodies coming with gunshot shelling or bullets.

Results from this study share some similar features of combat trauma as well as differences as compared with data from case reports, larger series of combat patients, and review papers [1, 9, 10, 17, 19, 31]. Similar to other researchers, we evaluated the severity of trauma by using various injury scales [9, 31]. This study also confirmed the utility of minimally invasive techniques such as VATS and laparoscopy for patients with severe gunshot injury, which is confirmation of our previously published cases [2, 24]. Our approach to using VATS during the war at Role 2 hospital is in line with Swiech et al., who applied a similar approach in Role 2 hospital in war theaters in Afghanistan and West Africa [30].

We have shown lethal outcome in 1 (3%) patient, which is in contrast with Morrison et al. who investigated a cohort of 27 combat patients with thoracoabdominal injury, showing lethal outcomes in 9 (33%) patients, Propper et al. demonstrated 13% and Kotwal et al. 38.5% of lethal cases in large series [9–11]. Other studies showed lethal cases in 3% of injured with thoracic penetrating trauma [32]. We consider the lower rate of deaths among injured in our cohort could be related to the “golden hour” policy for transportation to the hospital at Role 2, as we also have shown in our previous study [10, 32]. On the other hand, a higher proportion of force fatalities was shown in a smaller cohort as compared to others, which might affect our results. Still, results from our study indicate the importance of the “golden hour” policy to evacuate patients to the appropriate echelon (Role) of medical care within 1 h immediately after the casualty [1, 2, 5, 13, 17, 18, 33]. Similar to others and using our own experience since 2014, we have also demonstrated the utility of perfusion index for evacuation of hemodynamics, as well as using PI within the measures of damage control surgery and including damage control resuscitation (PI > 4 units were considered for the stable hemodynamics) [1, 4, 15, 19, 34, 35]. In this study, we have also demonstrated the utility of combining damage control surgery and damage control resuscitation as an integrated approach for patients in extreme conditions, allowing to avoid delays in surgical treatment and improving survival of the patients, which is also typical for combat patients [11, 15, 23].

## Conclusions

To sum up, abdominal and thoracoabdominal gunshot injuries are associated with critical and severe conditions of the patients due to the application of high-energy weapons in the ongoing war. Both abdominal and thoracoabdominal gunshot injuries might be managed at Role 2 hospitals by using minimally invasive technologies such as laparoscopy and video-assisted thoracoscopy (VATS) accompanied by surgical magnetic tools for better identification and removal of shells or bullet fragments with ferromagnetic properties. Damage control surgery and damage control resuscitation must be applied for patients in critical and severe conditions. By using Ukraine’s experience, this study might be considered as an evidence to update of current military medicine NATO guidelines for the application of minimally invasive surgery in deployed hospitals.

## Data Availability

All data generated or analyzed during this study are included in this published article.
